# Integrative analysis reveals ncRNA-mediated molecular regulatory network driving secondary hair follicle regression in cashmere goats

**DOI:** 10.1186/s12864-018-4603-3

**Published:** 2018-03-27

**Authors:** Guangxian Zhou, Danju Kang, Sen Ma, Xingtao Wang, Ye Gao, Yuxin Yang, Xiaolong Wang, Yulin Chen

**Affiliations:** College of Animal Science and Technology, Northwest A&F University, Yangling, 712100 China

**Keywords:** Hair follicle, lncRNA, miRNA, Skin, *Capra hircus*

## Abstract

**Background:**

Cashmere is a keratinized product derived from the secondary hair follicles (SHFs) of cashmere goat skins. The cashmere fiber stops growing following the transition from the actively proliferating anagen stage to the apoptosis-driven catagen stage. However, little is known regarding the molecular mechanisms responsible for the occurrence of apoptosis in SHFs, especially as pertains to the role of non-coding RNAs (ncRNAs) and their interactions with other molecules. Hair follicle (HF) degeneration is caused by localized apoptosis in the skin, while anti-apoptosis pathways may coexist in adjacent HFs. Thus, elucidating the molecular interactions responsible for apoptosis and anti-apoptosis in the skin will provide insights into HF regression.

**Results:**

We used multiple-omics approaches to systematically identify long non-coding RNAs (lncRNAs), microRNAs (miRNAs) and mRNAs expressed in cashmere goat skins in two crucial phases (catagen vs. anagen) of HF growth. Skin samples were collected from three cashmere goats at the anagen (September) and catagen (February) stages, and six lncRNA libraries and six miRNA libraries were constructed for further analysis. We identified 1122 known and 403 novel lncRNAs in the goat skins, 173 of which were differentially expressed between the anagen and catagen stages. We further identified 3500 gene-encoding transcripts that were differentially expressed between these two phases. We also identified 411 known miRNAs and 307 novel miRNAs, including 72 differentially expressed miRNAs. We further investigated the target genes of lncRNAs via both cis- and trans-regulation during HF growth. Our data suggest that lncRNAs and miRNAs act synergistically in the HF growth transition, and the catagen inducer factors (TGFβ1 and BDNF) were regulated by miR-873 and lnc108635596 in the lncRNA-miRNA-mRNA networks.

**Conclusion:**

This study enriches the repertoire of ncRNAs in goats and other mammals, and contributes to a better understanding of the molecular mechanisms of ncRNAs involved in the regulation of HF growth and regression in goats and other hair-producing species.

**Electronic supplementary material:**

The online version of this article (10.1186/s12864-018-4603-3) contains supplementary material, which is available to authorized users.

## Background

Cashmere goats are the main livestock breed used for the production of both cashmere and meat [1]. Cashmere has an important status in the textile industry due to its high economic value [2]. Improvement the quantity and quality of cashmere becomes an important breeding goal, and molecular breeding is a convenient way to locate functional genes that increase fiber production [3, 4]. Preventing or delaying hair loss is a matter of some priority. Hair loss and production mechanisms remain incompletely explained, though there have been several studies involving hair regeneration in humans or other model animals [5, 6].

Cashmere is produced by the secondary hair follicles (SHFs), which exhibit an annual periodicity, undergoing anagen (growth), catagen (regression), and telogen (resting) phases annually. The majority of the time spent in each cycle is occupied by anagen, while catagen can have a critical effect on telogen or even the complete cycle. The basis for HF involution rests in the unique follicular epithelial and mesenchymal elements, as well as other cells type (adipocytes) intercellular molecules communication [7, 8]. Some of the molecular signals involved in HF regression process have been determined, including fibroblast growth factor (FGF), transforming growth factor-β (TGF-β), tumor necrosis factor-β (TNF-β), Wnt, sonic hedgehog (SHH), neurotrophins (NT), and homeobox proteins [7, 9–13].

HF regression is not only associated with the regulation of HF-structured cells but is also affected by other types of cellular changes in the skin environment. Whole-transcriptome sequencing could provide new insights into the molecular regulatory mechanisms of the HF cycle and the interactions among HF cells. The functions of microRNAs (miRNAs) and long non-coding RNAs (lncRNAs) have been extensively reported in livestock, such as sheep and goat [14, 15], which played an important regulatory role in biological processes such as cell proliferation, differentiation, and apoptosis. MiRNAs are widely reported in animals and expressed in a temporal and spatial manner, playing an important role in hair follicle development and cycle [16–19]. We previously found that miRNAs are widely expressed in the skins of cashmere goats and that their expression levels were altered across the different HF cycling phases [16, 20, 21]. Specifically, the expression of miR-31 was significantly higher in the growth phase than in the regression phase; this miRNA targets the regulation of *KRT16*, *KRT17*, *DLX3*, and *FGF10*, thus affecting hair growth [22]. MiR-214 controls skin and HF development by modulating the activity of the Wnt pathway [23]. MiR-22 has been shown to be associated with HF degeneration and inhibits the expression of transcription factors such as *DLX3*, *FOXN1*, and *HOXC13* [24]. Thus, miRNAs play an indispensable regulatory role in various biological processes during the HF cycle and in the HF transitions to other stages.

Other non-coding RNAs (ncRNAs), such as lncRNAs, are essential for the regulation of hair growth and the HF cycle, though the functions of the lncRNAs involved in the HF cycle remain unclear. The expression of lncRNAs in mouse dermal papilla cells (DPCs) changes with subsequent passage generations, indicating that lncRNAs are related to dermal papilla (DP) characteristics [25]. LncRNAs have been found to be associated with hair growth, playing an important role in the development of SHFs in sheep [15]. The lncRNA PlncRNA-1 regulates the proliferation and differentiation of HF stem cells through the TGFβ1-mediated Wnt/β-catenin signaling pathway [26]. The expression of lncRNA-H19 changes according to the growth phase of goat SHFs [27]. Overall, lncRNAs as well as miRNAs play an important role in the regulation of HF growth and development.

Despite this progress, the regulation of hair cycling in mammals is complex, and there may be other regulatory channels involved. Previous studies have reported that lncRNAs act as regulatory genes that compete with miRNAs [28] to not only directly inhibit mRNA expression but also bind miRNAs to regulate mRNA expression. In this study, we aimed to elucidate the molecular mechanism of HF regeneration by determining the expression levels of mRNAs, lncRNAs, and miRNAs and their corresponding relationships in the skin microenvironment.

## Methods

### Samples

Three two-year-old female Shanbei Cashmere goats with unrelated genetic background were used in this study. Skin samples were biopsied at mid-September and mid-February, as previously described [29]. To minimize animal suffering, procaine was used for local anesthesia. The goats were sampled from the Shanbei Cashmere Goat Farm of Hengshan, Yulin, China (located at 37°21′–38°14′ N and 108°56′–110°01′ E), being raised in the same environment. Dorsal skin samples were collected from between ribs 12 and 13. Each skin sample, about 1 cm^2^, was cut into pieces and then stored in an RNA/DNA sample protection reagent (Takara, Dalian, China), immediately. Samples were transported in dry ice and stored at − 80 °C for total RNA extraction. All sampling procedures in this experiment were in accordance with approved guidelines of the Animal Care and Use Committee of the Northwest A&F University (Approval ID: 2014ZX08008–002).

### Total RNA isolation, library preparation, and sequencing

Total RNA was extracted using an Eastep® Super Total RNA Extraction Kit (Promega, Shanghai, China), according to the manufacturer’s instructions. We obtained two libraries from each sample: a lncRNA library and a miRNA library. The lncRNA library was prepared following a previous description [30], and library quality was assessed on the Agilent Bioanalyzer 2100 system. Libraries were sequenced on an Illumina HiSeq 4000 platform as 150-bp paired-end reads. Small RNA libraries were constructed and sequenced following a previous description [30], and the libraries were sequenced on an Illumina HiSeq 2500 platform using single-end reads.

### Quality control, annotation, and expression levels

Raw reads from lncRNA libraries were routinely processed using a Perl script. In this step, adapters, reads containing over 10% Ns, and low-quality reads (> 50% of bases with Phred scores < 5%) were removed. The Phred score (Q20, Q30) and GC content of the clean data were calculated. All subsequent analyses were based on the high-quality data. The goat (*Capra hircus*) reference genome and gene annotation files (Ver. ASM170441v1) were downloaded from the National Center for Biotechnology Information (NCBI, https://www.ncbi.nlm.nih.gov/). An index of the goat reference genome was built using Bowtie2 v2.3.1 [31], and paired-end clean reads were aligned to the reference genome using TopHat2 v2.1.1 [32]. Cufflinks v2.1.1 [33] was used to analyze gene patterns. lncRNAs were identified based on their structures and the fact that they do not encode proteins; specifically, the following five criteria were used [34]: 1) transcripts must contain no fewer than 2 exons; 2) the transcript length must be more than 200 bp; 3) screening the known lncRNAs and the unknown transcript left for the following analysis; 4) quantification of each transcript must be no less than 0.5; 5) remaining unknown transcripts that potentially encoded proteins were assessed. Tophat2 was run with ‘–library-type fr-firststrand’, and Cufflinks was run with ‘min-frags-per-transfrag = 0’, while other parameters were set to the defaults.

Raw reads from small RNA libraries were first processed using custom Perl and Python scripts, and redundant regions were removed. Then, we chose a certain range of length from clean reads for all the downstream analyses, and the annotation was performed following previously described methods [35] with miRBase21.0 used as a reference. Cuffdiff (v2.1.1) [33] was also used to calculate the fragments per kb per million reads (FPKM) for both lncRNAs and coding genes in each sample. MiRNA expression levels were estimated by transcripts per million (TPM) as previously described [35]. For biological replication, transcripts, genes, or miRNAs with a *P*-value of < 0.05 were considered differentially expressed between the two groups of goats. DEseq2 was used to analyze all differential expression experiments.

### Quantitative real-time PCR (qPCR) validation

Total RNAs were extracted from adult goat skin samples from the two groups and used for qPCR analysis. First strand cDNA was synthesized using the Thermo Scientific RevertAid First Strand cDNA Synthesis Kit (#K1622, Thermo Scientific, USA) according to the manufacturer’s instructions and was then subjected to quantification using a standard SYBR Premix Ex Taq (Tli RNaseH Plus) kit (#RR420A, Takara, China) on the Bio-Rad CFX96 Real-Time PCR Detection System with β-actin as an endogenous control. The qPCR procedure was carried out according to the instructions of the reagent kit: pre-denaturation at 95 °C for 30 s, followed by 41 cycles of denaturation at 95 °C for 5 s, annealing at 60 °C for 30 s, and extension at 72 °C for 30 s. The primers used for this experiment are listed in Additional file 1. Biological and technical replication was performed in triplicate for each sample. Relative gene expression was calculated using the 2^-ΔΔCt^ method and quantified relative to β-actin. Student’s *t*-tests were used for statistical analysis, and a *P*-value < 0.05 was considered to be significant. Values are expressed as means ± SD, * *P* < 0.05, ** *P* < 0.01.

### GO and KEGG pathway analysis

Gene ontology (GO) enrichment analysis of DE RNAs or lncRNA target genes was conducted with the GOseq R package, with a correction for gene length bias. GO terms with corrected *P*-values < 0.05 were considered significantly enriched DE genes. Kyoto Encyclopedia of Genes and Genomes (KEGG) database is a resource for understanding the high-level functions and utilities of a biological system, such as a cell, organism, or ecosystem, from molecular-level information, especially large-scale molecular datasets generated by genome sequencing and other high-throughput experimental technologies (http://www.genome.jp/kegg). We used KOBAS software to assess the statistical enrichment of DE genes or lncRNA target genes in KEGG pathways. Directed acyclic graph (DAG) is a graphical display of DE genes GO enrichment analysis results, and DAGs were drawn in the biological process (BP), cellular component (CC) and molecular function (MF), respectively.

### Correlation and co-expression/co-localization analysis

Co-expression analysis was based on calculating the Pearson correlation coefficients (PCCs) between coding genes and noncoding transcripts according to their expression levels. An absolute value of the parameter PCC ≥ 0.95, *P*-value < 0.01, and false discovery rate (FDR) < 0.01 was used for identifying genes for further analysis. For identifying cis-regulation, lncRNAs that act on neighboring target genes were investigated. We searched for coding genes 10 k/100 k upstream and downstream of each lncRNA and then analyzed their functions. For identifying trans-regulation, lncRNAs and target genes were identified based on their expression levels. We calculated the expression level correlation between lncRNAs and coding genes using custom scripts.

### Competing endogenous RNAs (CeRNAs) network analysis

To reveal the roles and interactions of ncRNAs and mRNAs in the HF growth cycle, we constructed ncRNA regulatory networks. Those lncRNAs and mRNAs whose expression levels were meaningfully correlated were included in this analysis. Potential miRNA response elements were searched for the sequences of lncRNAs and mRNAs, and we identified overlaps in predicted miRNA seed sequence binding sites and lncRNAs binding sites in the target mRNA as part of the lncRNA–miRNA–mRNA interaction. The miRNA binding sites were predicted by miRBase (http://mirbase.orghttp://mirbase.org), while the miRNA–mRNA interactions were predicted by Targetscan (http://www.targetscan.org/). The interaction network was built and visually displayed using Cytoscape software based on the screening of lncRNA–miRNA–mRNA pairs.

## Results

### Morphological features of goat skins at different stages

**Fig. 1:**
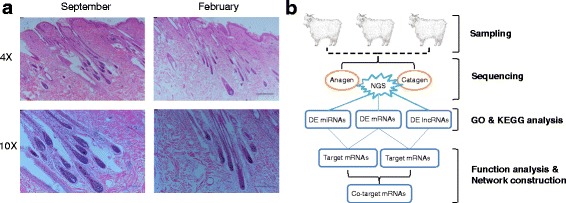
H&E staining of goat skins and overall study design. **(a)** H&E staining results of goat skins in the anagen and catagen phases. Red arrows show the location of hair dermal papilla. **(b)** Schematic workflow of the experimental design of this study

To examine the morphological differences in the growth to regression stages, we collected cashmere goat skin samples at anagen (September) and catagen (February). These time points were determined according to HF morphological analysis and fiber growth features. Hematoxylin and eosin (H&E) staining indicated clear differences in the hair bulbs of anagen and catagen goat skins. Specially, the sharp/size of DPs had a great change and their appearance were atrophying in February, while they were plump in September (Fig. 1a). These results indicate that a series of biological processes occur in goat skins throughout the year, and this further guaranteed that the proper samples were collected for further analysis. Therefore, a comprehensive whole-transcriptome sequencing analysis was used to systematically investigate the role of genes and ncRNAs in the biological processes of the HF transition from the anagen to the catagen stage (Fig. 1b).

### Identification of lncRNAs and miRNAs in goat skin

An average of 183,914,710 raw reads were produced on the Illumina HiSeq 4000 platform for each sample. After filtering out adaptor-related and low-quality sequences and those containing Ns, we obtained 176,158,389 clean reads, accounting for 26.4 Gb of clean sequence for each sample. Subsequently, we classified and mapped the clean reads to the latest goat reference genome assembly (ARS1, https://www.ncbi.nlm.nih.gov/assembly/GCF_001704415.1). The output data, raw read classification, and mapping region for each sample are summarized in Additional file 2 (Tables S1–S2) and Additional file 3: Figure S1, Additional file 3: Figure S2). We identified lncRNAs by characteristics that distinguish them from other RNAs (coding mRNAs, tRNAs, rRNAs, snRNAs, snoRNAs, pre-miRNAs, and pseudogenes), including the use of five rigorous criteria and screening tools for identifying potential coding sequences (see Additional file 3: Figure S3a). We identified 45,638 transcripts, 1122 known lncRNAs, 403 novel lncRNAs (Additional file 3: Figure S3b), and 350 transcripts of uncertain coding potential (TUCP). A list of these and their expression levels is provided in Additional file 4. For miRNA-seq, we obtained more than 28.6 M clean reads, accounting for 1.43 Gb of clean sequence for each sample (Additional file 2: Table S3). We identified 411 known miRNAs and 307 novel miRNAs (Additional file 5). A summary of the identified RNAs is provided in Table 1, and those determined to be differentially expressed (DE) between the anagen and catagen stages were used for further analysis.

**Table 1:** Summary of identified genes and ncRNAs

ncRNA	#Known	#Novel	#Known different transcripts	#Novel different transcripts
mRNAs	43,763	350	3500	87
lncRNAs	1122	403	107	66
miRNAs	411	307	55	17

### Differentially expressed mRNA, lncRNA, and miRNA profiles

**Fig. 2:**
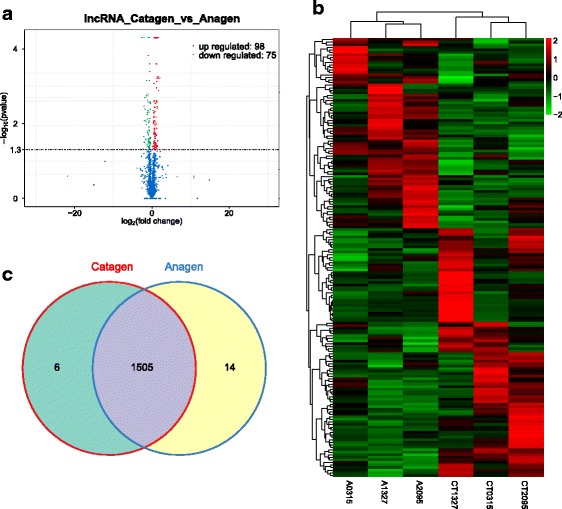
LncRNA expression profile changes in goat skins. **(a)** Volcano plot indicating up- and down-regulated lncRNAs in the catagen stage compared with the anagen stage. **(b)** Heat map of lncRNAs showing hierarchical clustering of altered lncRNAs in the catagen stage compared with the anagen stage. Up- and down-regulated genes are in red and green, respectively. **(c)** Venn diagram showing the number of overlapping lncRNAs in the catagen and anagen stages

**Fig. 3:**
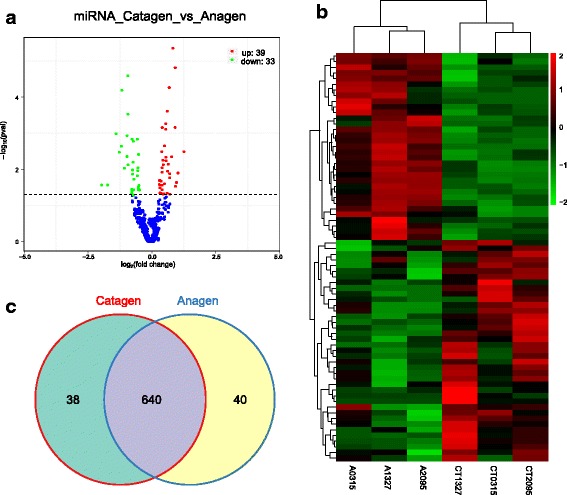
mRNA expression profile changes in goat skins. **(a)** Volcano plot indicating up- and down-regulated mRNA transcripts in the catagen stage compared with the anagen stage. **(b)** Heat map of mRNA transcripts showing hierarchical clustering of altered mRNA transcripts in the catagen stage compared with the anagen stage. Up- and down-regulated genes are in red and green, respectively. **(c)** Venn diagram showing the number of overlapping mRNA transcripts in the catagen and anagen stages

**Fig. 4:**
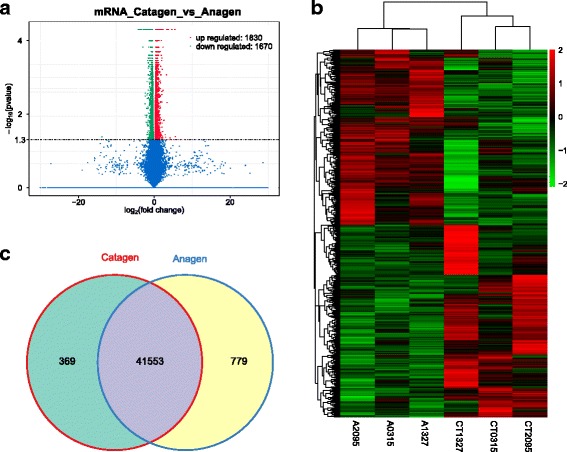
MiRNA expression profile changes in goat skins. **(a)** Volcano plot indicating up- and down-regulated miRNAs in the catagen stage compared with the anagen stage. **(b)** Heat map of mRNA transcripts showing hierarchical clustering of altered miRNAs in the catagen stage compared with the anagen stage. Up- and down-regulated genes are in red and green, respectively. **(c)** Venn diagram showing the number of overlapping miRNAs in the catagen and anagen stages

**Fig. 5:**
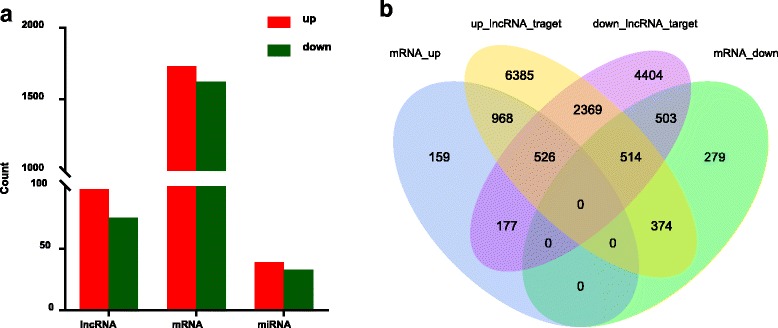
Count of relative ncRNAs and mRNAs in goat skins. **(a)** Histogram showing the number of up- and down-regulated ncRNAs and miRNAs in goat skins. **(b)** Venn diagram showing the number of overlapping targeted mRNAs of up-regulated lncRNAs, targeted mRNAs of down-regulated lncRNAs, and up- and down-regulated mRNAs

To determine whether ncRNAs are involved in the HF regression process. DE ncRNAs and mRNAs from catagen and anagen stages were visualized using a volcano plot and clustering map, while overlapping RNAs expressed in the two groups were visualized using a Venn diagram. There were 173 DE lncRNAs comparing the catagen to the anagen, including 98 up-regulated and 75 down-regulated lncRNAs (Fig. 2a–b, Additional file 6). Similarly, there were 3500 DE mRNA transcripts (3357 genes) comparing the catagen to the anagen, including 1830 up-regulated and 1670 down-regulated transcripts (Fig. 3a–c, Additional file 7), and there were 72 DE miRNAs comparing the catagen to the anagen, including 39 up-regulated and 33 down-regulated miRNAs (Fig. 4a–c, Additional file 8). Fig. 5a shows a summary histogram of DE lncRNAs, mRNAs, and miRNAs, and the top 20 most significantly DE ncRNAs are provided in Table 2. We found that those lncRNAs (*LOC102190274*, *LOC108635596*, *LOC108635657*, *LOC108636746*, *LOC108635658*, *LOC102188339*, *LOC108635659,* and *LOC108635656*) are potential regulators. LncRNAs regulate the expression of target genes (mRNAs), and this can be demonstrated by co-localization and co-expression. Given that DE mRNAs could be directly or indirectly regulated by lncRNAs, we identified the overlap between lncRNA target genes and DE mRNAs for further analysis. Fig. 5b shows the target mRNAs of lncRNAs, based on co-localization and co-expression, and the overlap between these and DE mRNAs in a Venn diagram.

**Table 2:** The information of top 20 ncRNAs

Gene_id	Symbol	Catagen_FPKM	Anagen_FPKM	log2(foldchange)	pvalue	qvalue
lncRNAs						
108,636,076	LOC108636076	3.27552	2.18113	0.586645	5.00E-05	0.00453085
102,190,274	LOC102190274	69.5476	108.324	− 0.639287	5.00E-05	0.00453085
108,635,596	LOC108635596	496.322	332.291	0.578828	5.00E-05	0.00453085
XLOC_016760	XLOC_016760	0.679671	0.270519	1.32911	5.00E-05	0.00453085
108,635,657	LOC108635657	176.509	119.452	0.563313	5.00E-05	0.00453085
108,636,746	LOC108636746	349.081	2088.57	−2.58088	5.00E-05	0.00453085
XLOC_011770	XLOC_011770	1.64242	0.964027	0.768677	5.00E-05	0.00453085
XLOC_035762	XLOC_035762	0.522913	0.23768	1.13755	5.00E-05	0.00453085
102,191,729	LOC102191729	5.17834	2.00733	1.36721	5.00E-05	0.00453085
XLOC_022928	XLOC_022928	0.776793	0.328905	1.23986	5.00E-05	0.00453085
108,635,658	LOC108635658	439.096	261.643	0.746936	5.00E-05	0.00453085
108,637,983	LOC108637983	16.501	6.96008	1.24538	5.00E-05	0.00453085
102,188,339	LOC102188339	170.957	331.264	−0.954346	5.00E-05	0.00453085
108,635,659	LOC108635659	329.178	167.788	0.972233	5.00E-05	0.00453085
108,637,984	LOC108637984	12.7238	6.5125	0.966241	5.00E-05	0.00453085
XLOC_011150	XLOC_011150	0	0.388551	#NAME?	5.00E-05	0.00453085
106,502,102	LOC106502102	1.45025	0.539867	1.42563	5.00E-05	0.00453085
106,503,367	LOC106503367	2.82561	1.51684	0.897489	5.00E-05	0.00453085
108,635,656	LOC108635656	647.611	384.805	0.750998	5.00E-05	0.00453085
108,636,333	LOC108636333	0.393285	0.165964	1.24471	5.00E-05	0.00453085
miRNAs						
chi-miR-26b-5p	–	36,994.40529	20,106.24622	0.83827	4.28E-06	0.0012203
chi-miR-9-5p	–	584.8240442	293.1119614	0.92855	1.48E-05	0.0021027
chi-miR-296-3p	–	869.3498021	1740.745814	−0.93111	2.51E-05	0.0023833
chi-miR-126-3p	–	99,329.19052	60,171.26529	0.69324	5.28E-05	0.0035946
novel_902	–	91.47212143	233.4059548	−1.1671	6.31E-05	0.0035946
chi-miR-10a-5p	–	121,759.63	78,052.61543	0.61618	0.000239	0.011345
chi-miR-146b-3p	–	1952.842666	3969.328822	−0.92414	0.000291	0.011832
chi-miR-10a-3p	–	291.264755	195.0896059	0.55629	0.000538	0.017629
chi-miR-708-3p	–	4905.081724	3185.797639	0.59626	0.000575	0.017629
novel_184	–	104.1424806	50.34351654	0.92423	0.000677	0.017629
chi-miR-30a-5p	–	70,051.28439	52,086.13725	0.41857	0.00068	0.017629
novel_134	–	148.851035	322.3613492	−0.96263	0.001149	0.027286
chi-miR-411a-3p	–	121.9818919	218.432541	−0.76409	0.001434	0.029372
chi-miR-27b-5p	–	1603.481251	2398.470514	−0.55609	0.001443	0.029372
chi-miR-379-3p	–	192.3674291	329.4816033	−0.71425	0.001648	0.031316
chi-miR-100-5p	–	192,463.8305	133,343.7653	0.50892	0.002202	0.039218
chi-miR-145-3p	–	5533.228655	4168.91426	0.39802	0.003202	0.053682
chi-miR-380-3p	–	668.4528705	966.4892172	−0.50836	0.003804	0.060233
chi-miR-29b-3p	–	422.3496069	227.8073258	0.78513	0.004412	0.064847
chi-miR-126-5p	–	751.4526306	498.5027456	0.5589	0.004551	0.064847

**Fig. 6:**
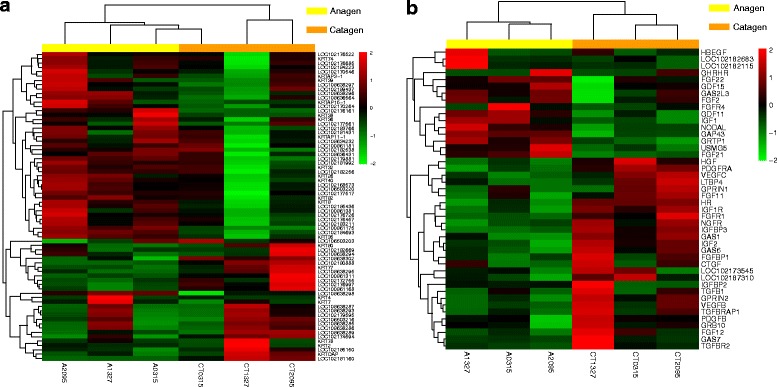
Heat maps for gene groups. **(a)** Heat map of differentially expressed *KRT* and *KRTAP* genes. **(b)** Heat map of differentially expressed growth factor-related genes

In comparing the RNAs expressed in the catagen and anagen phases, there were some genes of note. As keratin protein (KRT) and keratin-associated protein (KRTAP) are strongly associated with hair growth, we focused on these. We found that 74 *KRT* and *KRTAP* transcripts were differentially expressed between the catagen and the anagen stages (Fig. 6a). These genes are involved in driving the physiological characteristics of hair growth. KRT and KRTAP comprise the hair shaft and are an important part of cashmere. Most DE *KRT* and *KRTAP* genes (*KRT38*, *KRT4*, *KRTAP15–1*, *KRTAP13.1,* and *KRTAP3–1*) are involved in the construction of hair and were more highly expressed in the anagen than in the catagen stage. We also found that a few genes (*KRT2*, *KRTDAP*, *KRT77,* and *KRT80*) involved in epidermis keratinization were more highly expressed in the catagen stage. We also investigated DE growth factors or ligands associated with hair growth, and we found that the expression of growth arrest-specific genes (*GAS1*, *GAS6*, and *GAS7*) was higher in the catagen than in the anagen stage. We also found that growth factors and their receptors (*FGF22*, *FGF21*, *FGF2*, *GDF11*, *IGF1,* and *FGFR4*), as well as up-regulated skeletal muscle growth 5 homolog (*USMG5*) were more highly expressed in the anagen stage (Fig. 6b).

### Validation of mRNAs and lncRNAs

**Fig. 7:**
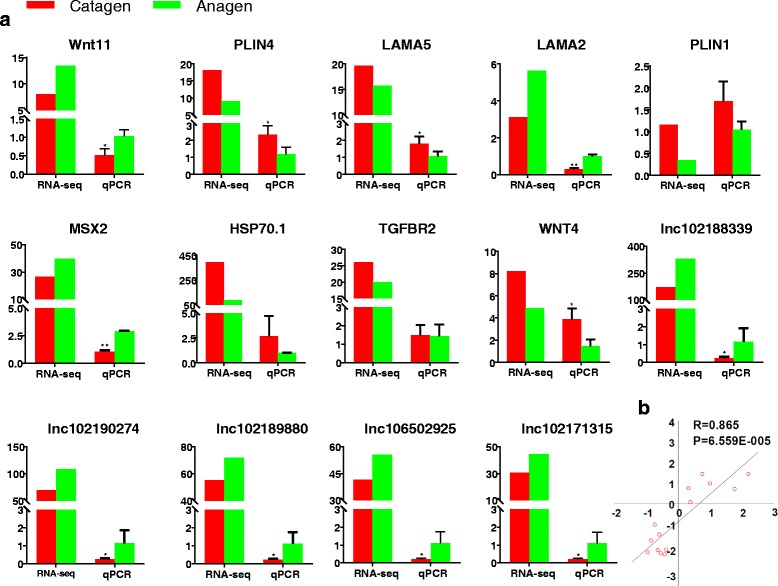
qPCR validation of RNA-seq. **(a)** Nine coding genes and five noncoding genes (lncRNAs are renamed “lnc” + the gene accession number). **(b)** Correlation analysis between qPCR and RNA-seq results. The *x*-axis indicates the fold change value log2^ratio(catagen/anagen)^ according to qPCR, and the *y*-axis indicates the log2^ratio(FPKMcatagen/anagen)^ value according to RNA-seq

To validate the reliability of the sequencing results, the expression changes of some mRNAs (*WNT11*, *PLIN4*, *LAMA5*, *LAMA2*, *PLIN1*, *MSX2*, *HSP70.1*, *TGFBR2,* and *WNT4)* and lncRNAs (*lnc10218839*, *lnc102190274*, *lnc102189880*, *lnc106502925,* and *lnc102171315)* were validated by qPCR (Fig. 7a). The expression levels of the genes as determined by the sequencing and qPCR methods are shown in Fig. 7b. These exhibited a correlation coefficient of 0.865 and *P*-value of 6.599E-05. The qPCR expression levels of all validated mRNAs and lncRNAs were consistent with the results obtained from HiSeq.

### Functional prediction of ncRNAs in the HF growth cycle

To explore the potential regulatory roles of lncRNAs in the HF growth cycle, we performed an integrated co-expression and co-localization network analysis of DE lncRNAs and mRNAs. We further selected genes in which the absolute value of the correlation was > 0.95 to predict the functions of lncRNAs using GO and KEGG analysis tools. Additional file 3: Figure S4a–c (Additional file 3) shows the BP, CC, and MF categories of the GO enrichment analysis based on the co-expression and co-localization of DE lncRNAs. The most significantly enriched BP terms were cellular metabolic process, metabolic process, and intracellular transport, and the noteworthy CC terms were protein complex, intracellular organelle part, and intermediate filament. The most significantly enriched MF terms were binding, protein binding, and ion binding (Additional file 3: Figure S4d). Additional file 3:Figure S5a–c (Additional file 3) shows the BP, CC, and MF categories of the GO enrichment analysis involving the overlapping co-expressed and co-localized DE lncRNA target genes, DE miRNA target genes, and predicted mRNAs. The most significantly enriched BP terms were biosynthetic process, metabolic process, and gene expression, and the most significantly enriched CC terms were intermediate filament, cytoskeleton, and protein complex. The most significantly enriched MF terms were binding, catalytic activity, and cytokine activity (Additional file 3: Figure S5d). Additional file 3: Figure S6a–c (Additional file 3) shown the BP, CC, and MF categories of the GO enrichment analysis of all DE genes. The most significantly enriched BP terms were protein import into nucleus, protein localization to nucleus, and protein targeting to nucleus, while the most significantly enriched CC terms were intermediate filament, intermediate filament cytoskeleton, and keratin filament. The most significantly enriched MF terms were cytokine activity, chemokine activity, and binding (Additional file 3: Figure S6d). These enriched categories of DE ncRNAs and their target genes showed potential values and will be the focus of future studies in HF regression.

An enriched scatter diagram of the candidate genes provides a graphic display of the KEGG enrichment analysis. The degree of KEGG enrichment is assessed by the richness factor, Q-value, and number of genes. We listed the top 20 most enriched pathways for DE mRNAs and lncRNA-miRNA-mRNAs by Q-value range (Additional file 3: Figure S7a–b). The most enriched pathways were disease pathways (pathways in cancer and systemic lupus erythematosus) and signaling molecules and interaction pathways (extracellular matrix (ECM)-receptor interaction). The ncRNA target genes exhibited KEGG enrichment patterns similar to those of the DE mRNAs. We divided the DE mRNA KEGG enrichment pathways into two categories: up-regulated and down-regulated (Additional file 3: Figure S7c–d). In the up-regulated category, the most significant pathways were pathways in cancer, cytokine–cytokine receptor interactions, adherens junctions, and the TNF signaling pathway. Most of these participate in cell apoptosis. Among the down-regulated pathways, the most significant pathways were metabolic pathways, oxidative phosphorylation, cell cycle, and ECM-receptor interactions. Additional KEGG pathways are provided in Additional file 9, and they deserve further analysis in the future.

### lncRNA-miRNA-mRNA networks

**Fig. 8:**
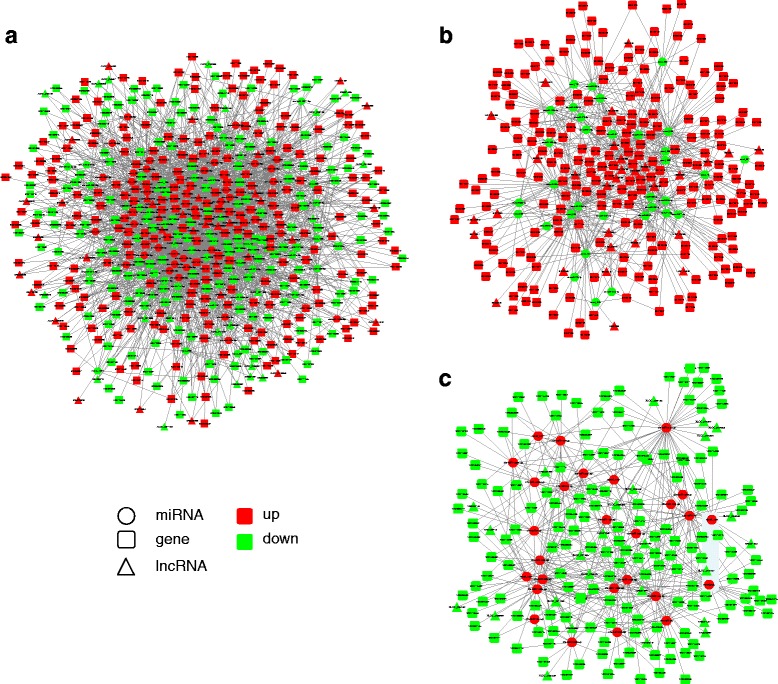
LncRNA–miRNA–mRNA regulatory networks in goat skin. Circles indicate miRNAs, squares indicate coding genes, and triangles indicate lncRNAs. Red indicates up-regulation, and green indicates down-regulation (catagen/anagen)

To explore the molecular mechanisms by which ncRNAs are involved in HF development and the HF cycle, we performed regulatory network analysis of ncRNAs and mRNAs in the two growth phases. MiRNAs have broadly regulatory functions involving RNA silencing and post-transcriptional regulation of gene expression, while lncRNAs also have extensive regulatory functions. We performed an integrated ncRNA and mRNA profiling analysis on the basis of their binding sites, with lncRNA-gene pairs and miRNA-gene pairs that share the same binding site being considered ceRNAs. We constructed lncRNA-miRNA-mRNA groups with the lncRNA as the decoy, miRNA as the center, and mRNA as the target (Fig. 8a). We then divided the ceRNA regulatory networks into two groups based on their gene expression patterns: up-down-up (Fig. 8b) and down-up-down (Fig. 8c). The results indicated that the expression of genes in the skin during HF regression is regulated by an ncRNA regulatory network that involves the action of ceRNAs. Our results (Table 3) present the regulatory relationships between ncRNAs and mRNAs in the processes of HF development and the HF cycle. Thus, further studies should be undertaken to better understand the mechanisms of HF development and cycle in the future.

**Table 3:** ncRNAs and their potential target genes involved in HF cycle

Genes	lncRNAs in cis	lncRNAs in trans	miRNAs
Transcription Factors			
STAT3	–	LOC102185409, LOC108635659, LOC108636392	-, chi-miR-10a-5p, chi-miR-1388-3p, chi-miR-125a-3p
TP63	–	LOC108634033	-, chi-miR-378-5p
SOX9	XLOC_031780	LOC108636392, LOC102185409, LOC108635596	-, chi-miR-125a-3p,chi-miR-30a-5p,chi-miR-141
MYC	LOC102186225	LOC108637984, LOC102185409, LOC108635656	-, chi-miR-338-3p, chi-miR-148a-3p, chi-miR-148a-3p
TFAP2	–	LOC108635596, LOC108636392, LOC108637897	-, chi-miR-141, chi-miR-141, chi-miR-148b-5p
LHX2	–	LOC108636746, LOC108636392	-, chi-miR-130b-3p, chi-miR-296-3p and chi-miR-125a-3p
MSX2	LOC102169517	LOC108635658, LOC108635659	-, chi-miR-1388-3p
FOXI3	–	LOC108636746	-, chi-miR-9-5p
HOXC13	–	LOC108635596, LOC102188339	-, novel_72 and chi-miR-141, chi-miR-873-5p
GATA3	–	LOC108635658, LOC108636746	-, chi-miR-1388-3p, novel_926
WNT/TGFB related			
WNT2	–	LOC106502925	-, chi-miR-29a/b-3p
WNT3	–	LOC108635656, LOC108636392	-, chi-miR-148a-3p, chi-miR-125a-3p
WNT4	LOC102189078	LOC108637984 and LOC102185409	-, chi-miR-338-3p
WIF1	–	LOC108636392	-, chi-miR-330-3p
DKK1	–	LOC108635596, LOC108636746 and LOC108636392	-, novel_72, chi-miR-9-5p
TGFBR2	–	LOC108635596 and LOC108636392	-, chi-miR-141
SAMD5	–	LOC108635656, LOC108635596	-, chi-miR-148a-3p, novel_72
TGFB1	–	LOC108635596, LOC108636746	-, chi-miR-873-5p, novel_926 and chi-miR-9-5p
BAMBI	–	LOC108635596, LOC108636746	-, chi-miR-141, novel_926
FGF/FGFR related			
FGF2	–	LOC108635596 and LOC102188339	-, chi-miR-873-5p
FGF11	–	LOC108637983 and LOC108637984	-, chi-miR-140-5p
FGF12	–	LOC108637984, LOC102185409	-, chi-miR-502b-5p, chi-miR-340-5p
FGF21	–	LOC108635596 and LOC102188339	-, chi-miR-873-5p
FGFBP1	–	LOC108636746	-, novel_926 and chi-miR-9-5p
FGFR1	–	LOC108635596 and LOC102188339	-, chi-miR-873-5p
Notch related			
DLL4	–	LOC106502925, LOC106502506,	-, novel_735, novel_1008
JAG2	–	LOC108636746, LOC108636392	-, chi-miR-9-5p, chi-miR-330-3p
ADAM17	–	LOC108635596, LOC108636392, LOC108637983	-, novel_72, chi-miR-125a-3p, chi-miR-26a/b-5p
HEY1	–	LOC102171315, LOC108637983	-, chi-miR-101-3p, chi-miR-378-3p and chi-miR-140-5p
DLK2	–	LOC108636746, LOC108636392	-, novel_926, chi-miR-125a-3p
DTX1	–	LOC108635659, LOC106502506	-, novel_996, chi-miR-25-5p
DTX3L	–	LOC108635656, LOC108635596, LOC108636746	-, chi-miR-148a-3p, chi-miR-873-5p, chi-miR-130b-3p
DTX4	–	LOC106502925, LOC108636392	-, chi-miR-29a/b-3p, novel_965
SHH related			
SUFU		LOC108635656, LOC108636746	-, chi-miR-148a-3p and chi-miR-873-5p, novel_926
PRKX		LOC108636392, LOC108637983 and LOC102185409	-, chi-miR-125a-3p, chi-miR-378-3p
GLI3		LOC108636392, LOC102171315	-, chi-miR-24-5p, chi-miR-23b-3p
BMP/TGFβ related			
BMP2	–	LOC108635659, LOC108636392	-, novel_996, novel_965 and chi-miR-125a-3p
TGFB1	–	LOC108635596, LOC108636746, LOC102188339	-, chi-miR-873-5p, novel_926 and chi-miR-9-5p, chi-miR-873-5p
TGFBRAP1	–	LOC108635656, LOC102188339	-, chi-miR-148a-3p and chi-miR-873-5p, chi-miR-873-5p
TGFBR2	–	LOC108635596 and LOC108636392, 102,171,315	-, chi-miR-141, chi-miR-23b-3p

## Discussion

Skin, as the first line of defense consisting of diverse cells (adipocyte and fibroblast) and affiliate organs (HFs and sweat glands), covers the entire body. HFs are an important component of the skin, consisting of primary HFs and SHFs. SHFs account for over 90% of the two types of HFs in cashmere goats, and they produce fine fibers in a rigorous annual cycle that involves anagen, catagen, and telogen [36]. During catagen, the DPs of SHFs shrink, and therefore, it is presumed that there is strong apoptosis signaling in DPs and anti-apoptosis singling in adjacent DPs. While seven theories have been proposed to explain the HF cycle (epithelial theory, papilla morphogen theory, bulge activation theory, resonance theory, oscillating signal theory, inherent embryonic cycle theory, and inhibition-disinhibition theory) [37], the underlying mechanism for regression remains unknown. In this study, we performed an ncRNA and coding RNA profiling analysis by comparing the DE RNAs of the anagen and the catagen phases, providing novel insights into the mechanism of the HF cycle.

We initially performed a histological analysis to show that the DPs of SHFs experienced atrophy in February and were full in September. This phenomenon was consistent with expectations and provided the basis for the subsequent analysis. Next, we performed whole-transcriptome sequencing and a comparative analysis of goat skins in different growth phases. Phenotypic appearance of HFs is affected by the expression of genes, which is regulated by various factors [38, 39]. The findings obtained from several studies have revealed the critical roles of ncRNAs in the hair growth cycle. Indeed, miRNAs have been frequently reported as regulators of skin and HF development [40], and there is also considerable evidence supporting the idea that lncRNAs play causal roles in the HF growth cycle [15, 25]. However, there was previously a lack of information regarding the role of ncRNAs in the HF cycle. In this study, we found that abundant RNAs, including lncRNAs, miRNAs, and coding mRNAs, were enriched in the skin and differentially expressed between the anagen and catagen phases. Some of these transcripts were differentially expressed in the skin at different growth phases, indicating that they play an important role in the regulation of biological processes, in particular those associated with apoptosis, anti-apoptosis, and growth processes [41–43]. Our results showed that a total of 173 lncRNAs, 72 miRNAs, and 3500 mRNA transcripts were significantly differentially regulated when comparing the catagen and anagen phases. These data indicate that many miRNAs are involved in HF development, and some of these miRNAs differ from those observed in previous research studies [16], although there were also some common miRNAs (miR-27a-5p, miR-99a-5p, miR-380-3p, and miR-9-5p) that deserve further attention. One reason for this discrepancy is the difference in analytical strategies. Another reason is that the same biological processes may be induced by a different combination of genes that coordinate with each other. In addition, the analysis of lncRNAs and their roles as ceRNAs provide new insight into these differences. Despite the fact that the functions of lncRNAs in HF development and the HF cycle are still poorly understood, the rhythmic expression of lncRNA genes indicates their casual roles in biological processes. Numerous lncRNAs, acting as decoys for miRNAs, have been found to play critical roles in biological functions such as apoptosis, proliferation, and differentiation [44–47].

Although we screened for DE ncRNAs from goat skins in different growth phases and a few ncRNAs were confirmed in the present study, the underlying mechanisms of ncRNAs in the HF cycle are poorly understood. We then analyzed DE ncRNAs-related gene functions and their corresponding pathways through GO and KEGG term enrichment analyses. Our data showed that the most significantly enriched pathway was pathways in cancer, a comprehensive pathway involving multiple cellular processes. Other cancer pathways, such as prostate cancer and proteoglycans in cancer, as well as cytokine–cytokine receptor interaction, estrogen signaling pathway, adherens junction, and the Jak-STAT signaling pathway pointed to roles in cell growth, proliferation, and apoptosis/anti-apoptosis. These pathways fully illustrate the balance between apoptosis and anti-apoptosis in the skin. The DPs of SHFs begin to atrophy while other cells experience accelerated growth and production of keratins to protect against environmental stress. There were also some lipid metabolism and endocrine system KEGG pathways contributing to fat deposition as well as the hair growth cycle. The predicted functions of ncRNAs in the HF cycle, as determined by the GO and KEGG analyses, should be studied at a greater depth in future work.

The majority of fine hair growth slows down or ceases during catagen, including the production of KRT and KRTAP, which are indispensable structural components of hair. Most of DE *KRT* and *KRTAP* genes were down-regulated during catagen. Interestingly, we found that the expression levels of *KRT2*, *KRTDAP*, *KRT77,* and *KRT80* were higher during catagen. These genes mainly participate in epidermis keratinization, not in the production of hair components, indicating that epidermal cells experience extra growth during the catagen phase; this may be a defense mechanism against upcoming extreme environments (i.e., the cold winter). We also found that the expression of *GAS1* was higher during catagen than during anagen; this gene plays a role in growth suppression, blocking entry to S phase and preventing the cycling of cells [48]. This is consistent with what happens to the DPs of SHFs during catagen. Additionally, the expression levels of *FGF22*, *FGF21*, *FGF2*, *GDF11*, *IGF1*, *FGFR4,* and *USMG5* were lower during catagen, indicating that the growth of the entire skin slowed down, perhaps caused by the transition of SHFs from anagen to catagen. It is worth mentioning that *HSPH1*, *HSPA6*, *HSP70.1*, *LOC102178315* (heat shock 70 kDa protein 1B), and *HSPB1* were also highly expressed during catagen. These genes belong to the heat shock protein family and are involved in stress resistance, such as ensuring the correct folding of proteins or controlling the targeting of proteins for subsequent degradation [49–51]. These proteins would therefore be useful in preventing apoptosis, making a response to DPs adjacent organization. Next, we found that the expression levels of *AR* and *DKK1* were higher during catagen. *AR*, an androgen receptor, modifies the expression of the Wnt antagonist *DKK1* in DPs, preventing HF stem cell differentiation [52]. Thus, this is another factor associated with fatty acids or adipocytokines that might trigger a non-apoptotic DP cell death. Intradermal adipocytes are distributed around SHFs in the skin, making this hypothesis more relevant. We detected that the expression levels of *FASN* and *TRL4* were higher during catagen, and these two genes may also play a role in non-apoptotic cell death [53].

**Fig. 9:**
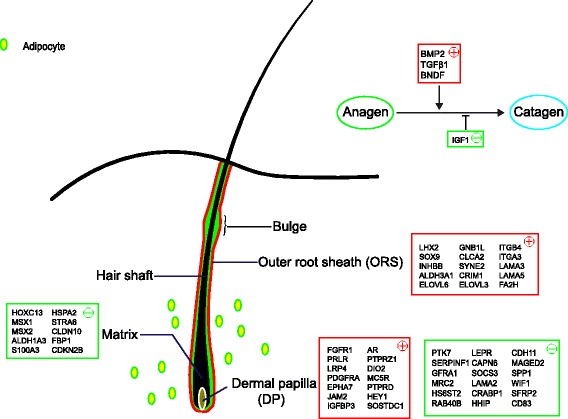
A proposed model for the microenvironment of the skin during the HF transition from anagen to catagen. Red squares indicate that expression of these genes is up-regulated during catagen, while green squares indicate down-regulation

Based on these lines of evidence, we propose a model (Fig. 9) for understanding what occurs in the skin during catagen, with gene locations based on previous studies [54]. Some evidence indicates that *BMP2*, *TGFβ1,* and *BDNF* could represent inducers of catagen, while *IGF1* could inhibit catagen [23]. HF stem cells return to quiescence via the action of BMPs (*BMP2*) and Wnt inhibitors, and their characteristics are maintained during HF regression by *LHX2*, *SOX2*, *ITGA3*, *ITGB4,* and other factors [55]. Meanwhile, the matrix undergoes apoptosis, and hair shaft formation slows. *HOXC13*, *MSX1*, *MSX2*, and other factors regulate the proliferation and differentiation of the hair matrix, and their expression were down-regulated during anagen in our study. Under the mediation of various genes, apoptosis and anti-apoptosis coexist among different cells in a single organism. The expression levels of *CASP8* and *MYC* are higher during catagen, contributing to apoptosis, while the increased expression levels of *BAG3*, *HSP70*, and *XIAP* during catagen may contribute to anti-apoptosis. *CASP14* is helpful for keratinization. The factors leading to the regression of HF still require further study and additional lines of evidence.

## Conclusions

In this study, we provided new insight into the molecular mechanisms driving the transition from anagen to catagen in HFs. We integrated analysis of lncRNAs, miRNAs, and mRNAs to determine their predicted roles and regulatory relationships in the HF cycle. We also provided a catalog of predicted ncRNAs in goat skin that will help to explain their regulatory roles in the goat HF cycle. In future studies, we plan to further examine the functions of these predicted DE ncRNAs, which may ultimately enable the full elucidation of the regulatory mechanisms associated with the goat HF cycle at the molecular level.

## Additional files

Additional file 1: Primers used in this study. (XLSX 10 kb)

Additional file 2: Summary data output for each sample (DOCX 17 kb)

Additional file 3: **Figure S1.** Raw reads classification for each sample (lncRNAs); **Figure S2.** Mapped region for each sample (lncRNAs);**Figure S3.** Novel lncRNAs filter. (a) Merged all sample transcripts and remove the chain direction uncertain transcript. Step1, the number of transcripts possessing no less than 2 exons; step 2, the number of transcripts possessing more than 200 bp length; step 3, the left number of transcripts getting rid of known lncRNAs; step 4, the left transcripts which FPKM ≥0.5; step 5, three coding potential screening tools (Coding-Non-Coding-Index, CNCI; Coding Potential Calculator, CPC and Pfam Scan, PFAM) applied to predict novel lncRNAs. (b) A venn diagram for step 5.;**Figure S4.** LncRNAs targeted genes GO analysis. (a–c) The significant molecular function, biological process and cellular component were enriched by lncRNAs targeted mRNAs in goat skins, and the DAG is the graphical display of GO enrichment results with candidate genes. (d) The number of genes in GO term were showed in histograph. **Figure S5.** GO analysis of lncRNA-miRNA target mRNAs. (a–c) The significant molecular function, biological process and cellular component were enriched by lncRNA-miRNA-mRNAs in goat skins, and the DAG is the graphical display of GO enrichment results with candidate targeted genes. (d) The number of genes in GO terms were showed in histograph. **Figure S6.** GO analysis of DE genes. (a–c) The significant molecular function, biological process and cellular component were enriched by DE mRNAs in goat skins, and the DAG is the graphical display of GO enrichment results with candidate targeted genes. (d) The number of genes in GO terms were showed in histograph. **Figure S7.** The top 20 KEGG pathways of hair cycle in skin. When the rich factor is greater, the Q-value is closer to zero, and the number of genes is greater, then the enrichment is more significant. (PDF 2925 kb)

Additional file 4: FPKM for total transcripts and novel transcripts information. (XLSX 5602 kb)

Additional file 5: TMP for total miRNAs and novel miRNAs information. (XLSX 108 kb)

Additional file 6: The information for DGE lncRNAs. (XLSX 25 kb)

Additional file 7: The information for DGE coding mRNAs. (XLSX 353 kb)

Additional file 8: The information for DGE miRNAs. (XLSX 14 kb)

Additional file 9: The enriched pathway terms. (XLSX 100 kb)

## Abbreviations

BP: Biological process
CC: Cellular component
ceRNAs: Competing endogenous RNAs
DEGs: Differentially expressed genes
DP: Dermal papilla
DPCs: Dermal papilla cells
FPKM: Fragments Per Kilobase of exon per Million fragments mapped
GO: Gene Ontology
HF: Hair follicle
KEGG: Kyoto Encyclopedia of Genes and Genomes.
KRT: Keratin protein
KRTAP: Keratin associated protein
LncRNA: Long noncoding RNA; miRNA, microRNA
MF: Molecular function
SHF: Secondary hair follicle
TMP: Transcript per million
TUCP: Transcripts of uncertain coding potential
USMG5: Skeletal muscle growth 5 homolog
