# Supplementary material for: Integrative analysis reveals ncRNA-mediated molecular regulatory network driving secondary hair follicle regression in cashmere goats
Source: BMC Genomics. 2018 Mar 27;19:222. doi: 10.1186/s12864-018-4603-3 (PMC5870523; doi:10.1186/s12864-018-4603-3)
Supplement: Supplementary file 2 — Summary data output for each sample (DOCX 17 kb) [file 12864_2018_4603_MOESM2_ESM.docx]

Table S1 Summary data output for each sample (LncRNA libraries)

| Sample name | Raw reads | Clean reads | clean bases | Error rate(%) | Q20(%) | Q30(%) | GC content(%) | NCBI ID |
| --- | --- | --- | --- | --- | --- | --- | --- | --- |
| A0315 | 196804348 | 192496462 | 28.87G | 0.02 | 97.3 | 92.75 | 52.1 | SRR6075308 |
| A1327 | 173243572 | 163963372 | 24.59G | 0.02 | 97.11 | 92.34 | 52.51 | SRR6075307 |
| A2095 | 190993966 | 182071572 | 27.31G | 0.02 | 96.84 | 91.78 | 53.14 | SRR6075306 |
| CT0315 | 181581056 | 173745444 | 26.06G | 0.02 | 96.81 | 91.67 | 51.26 | SRR6075305 |
| CT1327 | 171092966 | 163202582 | 24.48G | 0.02 | 96.95 | 92 | 52.32 | SRR6075310 |
| CT2095 | 189772354 | 181470904 | 27.22G | 0.02 | 97.25 | 92.73 | 52.33 | SRR6075309 |

Table S2 Raw reads classification for each sample (LncRNAs)

| Sample | total_reads | N% > 10% | low quality | 5_adapter_contamine | 3_adapter_null or insert_null | with ployA/T/G/C | clean reads |
| --- | --- | --- | --- | --- | --- | --- | --- |
| A0315 | 28978558 (100.00%) | 633 (0.00%) | 77671 (0.27%) | 1689 (0.01%) | 735455 (2.54%) | 31855 (0.11%) | 28131255 (97.08%) |
| A1327 | 28800300 (100.00%) | 597 (0.00%) | 69328 (0.24%) | 1155 (0.00%) | 505519 (1.76%) | 35402 (0.12%) | 28188299 (97.88%) |
| A2095 | 29047149 (100.00%) | 370 (0.00%) | 80009 (0.28%) | 1529 (0.01%) | 891008 (3.07%) | 28487 (0.10%) | 28045746 (96.55%) |
| CT0315 | 27529435 (100.00%) | 553 (0.00%) | 64141 (0.23%) | 579 (0.00%) | 319719 (1.16%) | 15945 (0.06%) | 27128498 (98.54%) |
| CT1327 | 29596412 (100.00%) | 390 (0.00%) | 79120 (0.27%) | 1089 (0.00%) | 617627 (2.09%) | 20445 (0.07%) | 28877741 (97.57%) |
| CT2095 | 28127102 (100.00%) | 605 (0.00%) | 66259 (0.24%) | 765 (0.00%) | 528837 (1.88%) | 19249 (0.07%) | 27511387 (97.81%) |

Table S3 Summary data output for each sample (miRNA libraries)

| Sample | Reads | Bases | Error rate | Q20 | Q30 | GC content | NCBI ID |
| --- | --- | --- | --- | --- | --- | --- | --- |
| A0315 | 28978558 | 1.449G | 0.01% | 98.83% | 98.01% | 50.65% | SRR6076964 |
| A1327 | 28800300 | 1.440G | 0.01% | 98.77% | 97.83% | 50.98% | SRR6076965 |
| A2095 | 29047149 | 1.452G | 0.01% | 98.88% | 98.07% | 50.77% | SRR6076962 |
| CT0315 | 27529435 | 1.376G | 0.01% | 98.85% | 98.04% | 50.55% | SRR6076963 |
| CT1327 | 29596412 | 1.480G | 0.01% | 98.89% | 98.09% | 50.28% | SRR6076960 |
| CT2095 | 28127102 | 1.406G | 0.01% | 98.82% | 97.96% | 50.78% | SRR6076961 |
