# Supplementary material for: Integrative analysis reveals ncRNA-mediated molecular regulatory network driving secondary hair follicle regression in cashmere goats
Source: BMC Genomics. 2018 Mar 27;19:222. doi: 10.1186/s12864-018-4603-3 (PMC5870523; doi:10.1186/s12864-018-4603-3)
Supplement: Supplementary file 3 — Figure S1. Raw reads classification for each sample (lncRNAs); Figure S2. Mapped region for each sample (lncRNAs);Figure S3. Novel lncRNAs filter. (a) Merged all sample transcripts and remove the chain direction uncertain transcript. Step1, the number of transcripts possessing no less than 2 exons; step 2, the number of transcripts possessing more than 200 bp length; step 3, the left number of transcripts getting rid of known lncRNAs; step 4, the left transcripts which FPKM ≥0.5; step 5, three coding potential screening tools (Coding-Non-Coding-Index, CNCI; Coding Potential Calculator, CPC and Pfam Scan, PFAM) applied to predict novel lncRNAs. (b) A venn diagram for step 5.;Figure S4. LncRNAs targeted genes GO analysis. (a–c) The significant molecular function, biological process and cellular component were enriched by lncRNAs targeted mRNAs in goat skins, and the DAG is the graphical display of GO enrichment results with candidate genes. (d) The number of genes in GO term were showed in histograph. Figure S5. GO analysis of lncRNA-miRNA target mRNAs. (a–c) The significant molecular function, biological process and cellular component were enriched by lncRNA-miRNA-mRNAs in goat skins, and the DAG is the graphical display of GO enrichment results with candidate targeted genes. (d) The number of genes in GO terms were showed in histograph. Figure S6. GO analysis of DE genes. (a–c) The significant molecular function, biological process and cellular component were enriched by DE mRNAs in goat skins, and the DAG is the graphical display of GO enrichment results with candidate targeted genes. (d) The number of genes in GO terms were showed in histograph. Figure S7. The top 20 KEGG pathways of hair cycle in skin. When the rich factor is greater, the Q-value is closer to zero, and the number of genes is greater, then the enrichment is more significant. (PDF 2925 kb) [file 12864_2018_4603_MOESM3_ESM.pdf]

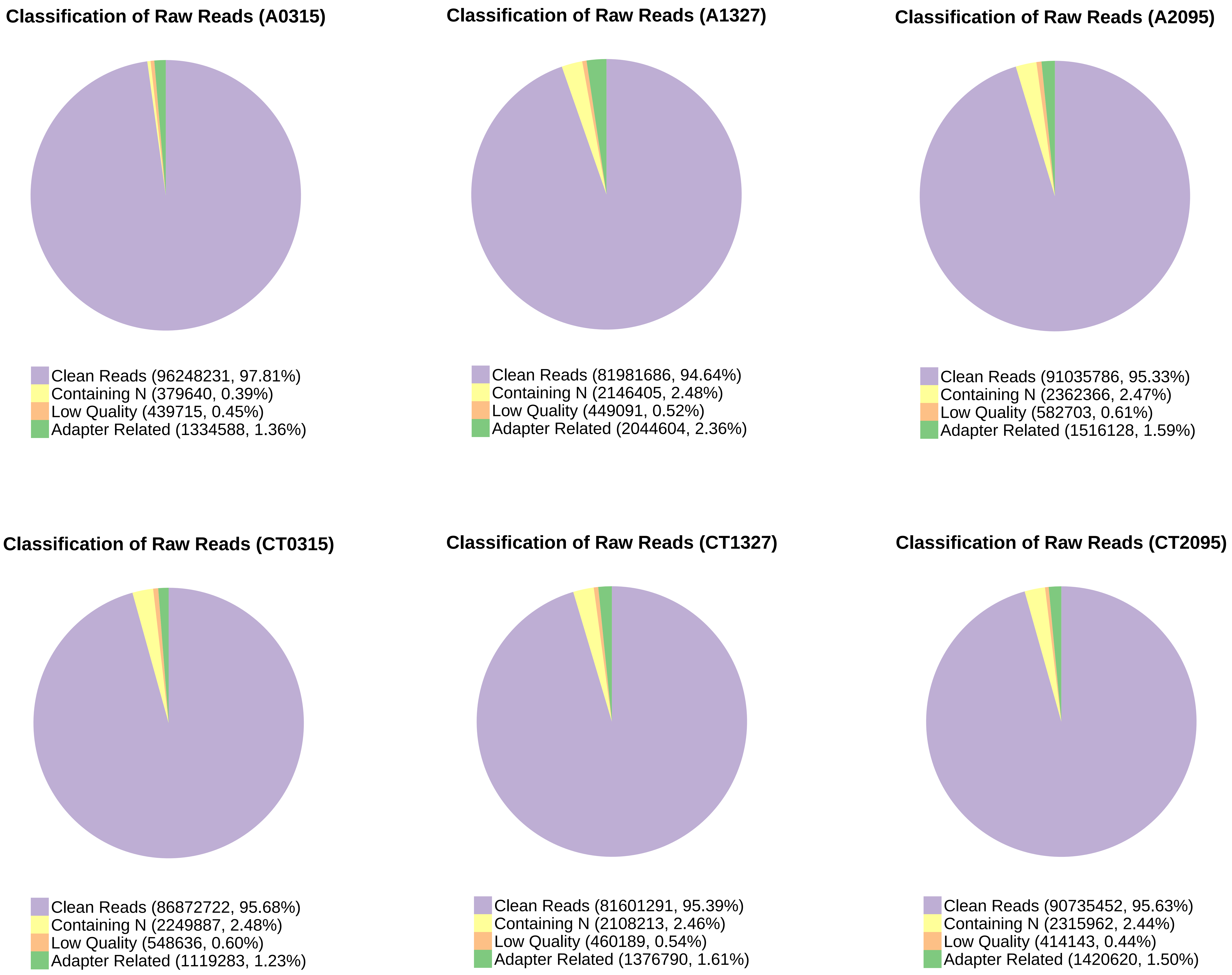

Figure S1 Raw reads classification for each sample

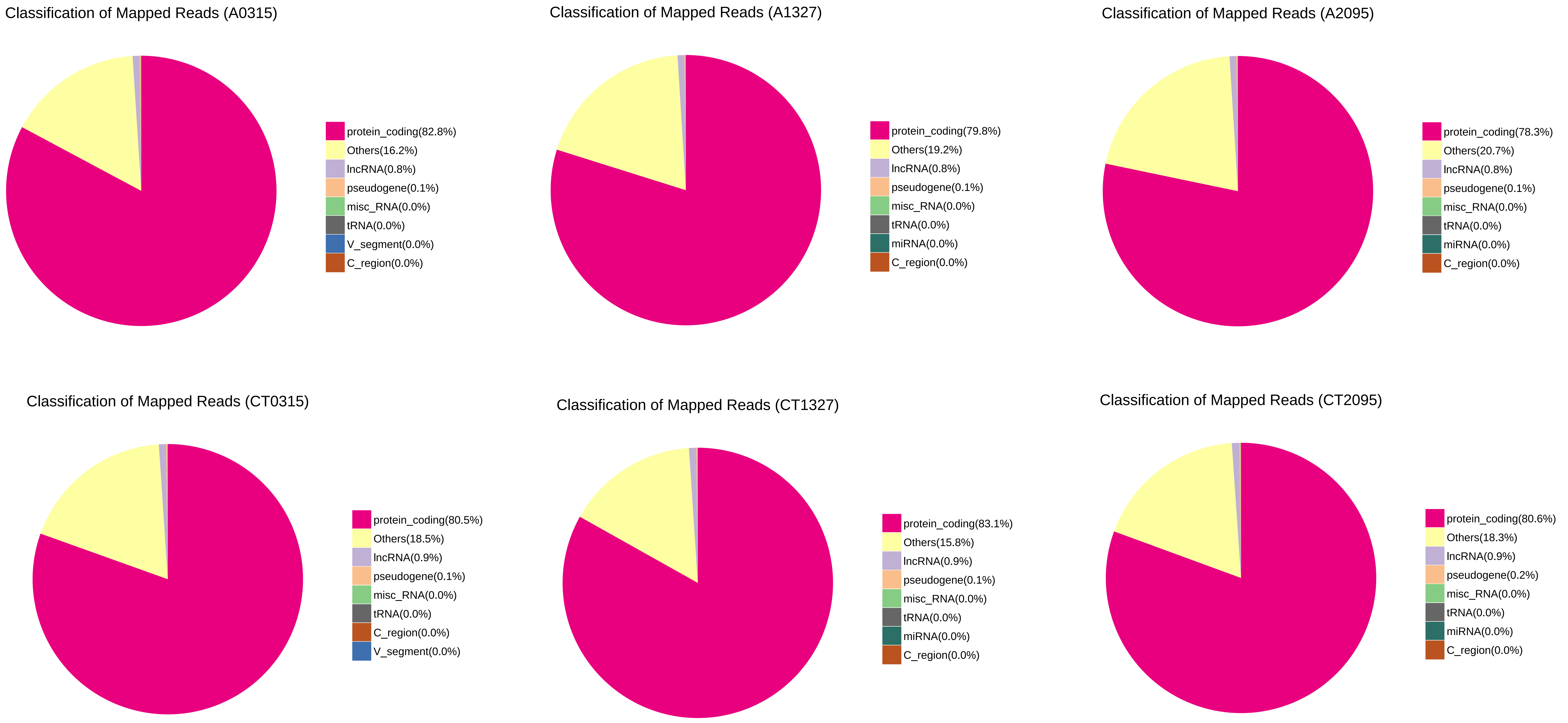

Figure S2 Mapped region for each sample

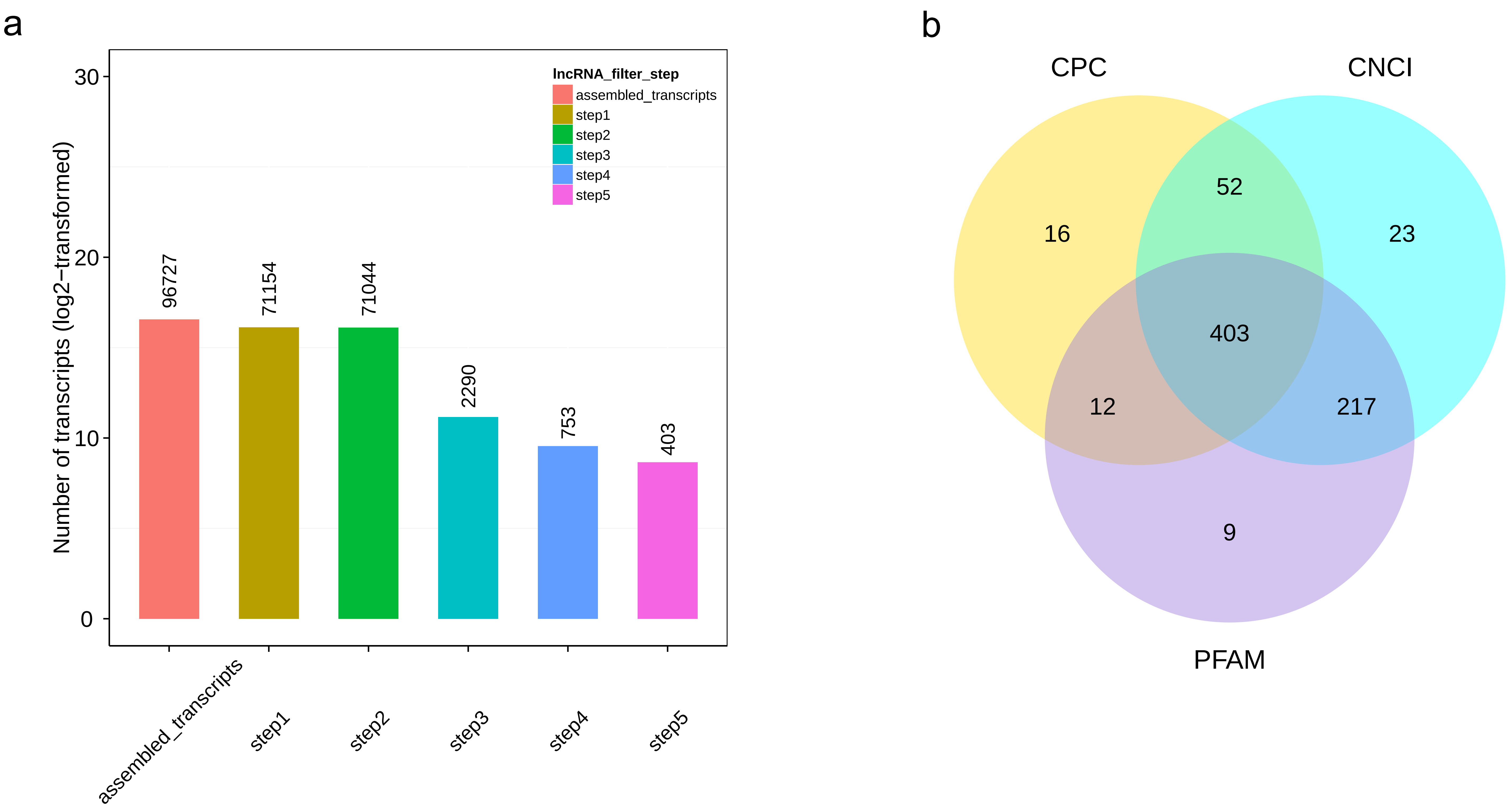

Figure S3. Novel lncRNAs filter. (**a**) Merged all sample transcripts and remove the chain direction uncertain transcript. Step1, the number of transcripts possessing no less than 2 exons; step 2, the number of transcripts possessing more than 200 bp length; step 3, the left number of transcripts getting rid of known lncRNAs; step 4, the left transcripts which FPKM ≥ 0.5; step 5, three coding potential screening tools (Coding-Non-Coding-Index, CNCI; Coding Potential Calculator, CPC and Pfam Scan, PFAM) applied to predict novel lncRNAs. **(b)** A venn diagram for step 5.

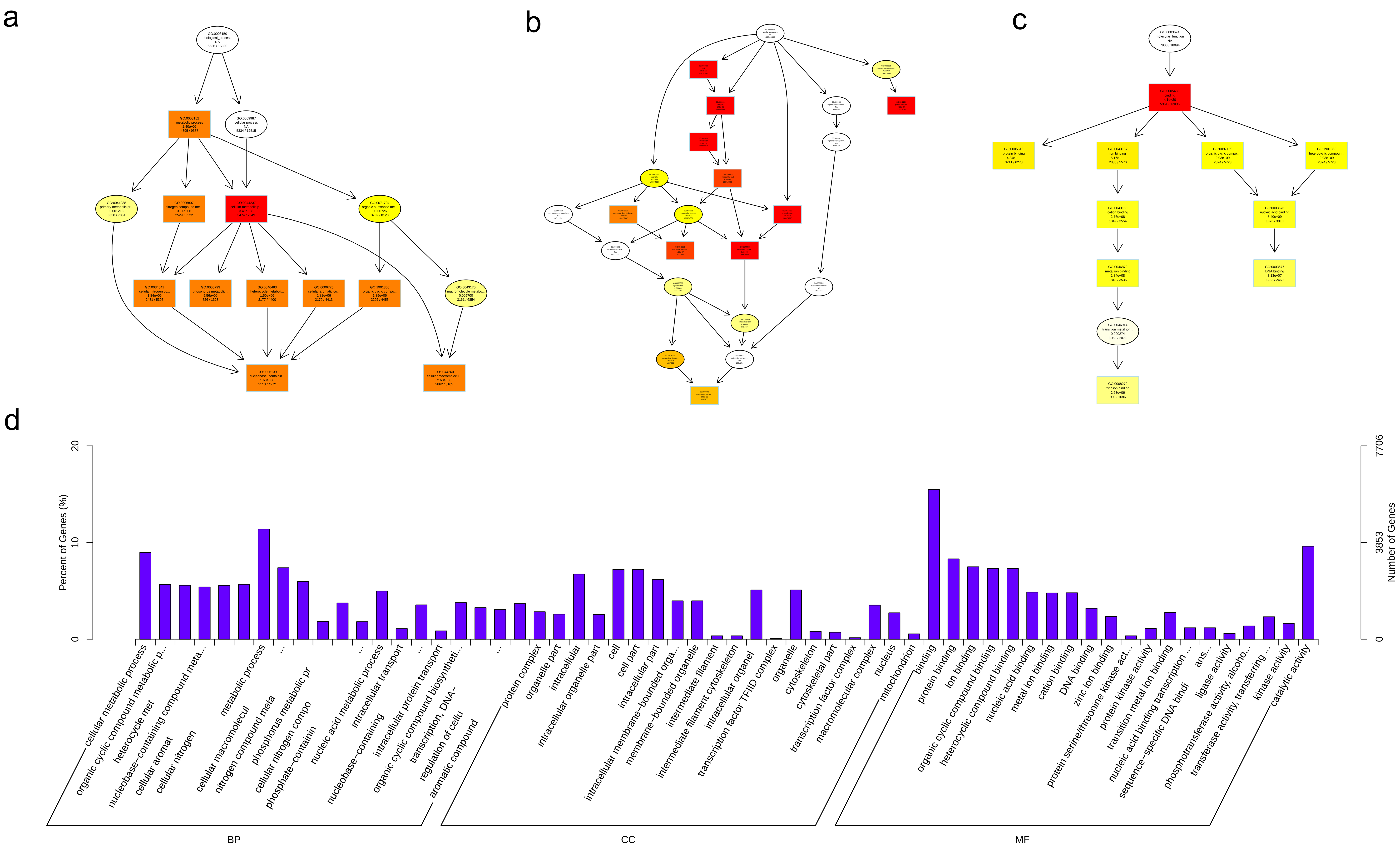

Figure S4. LncRNAs targeted genes GO analysis. (a–c) The significant molecular function, biological process and cellular component were enriched by lncRNAs targeted mRNAs in goat skins, and the DAG is the graphical display of GO enrichment results with candidate genes . **(d)** The number of genes in GO term were showed in histograph.

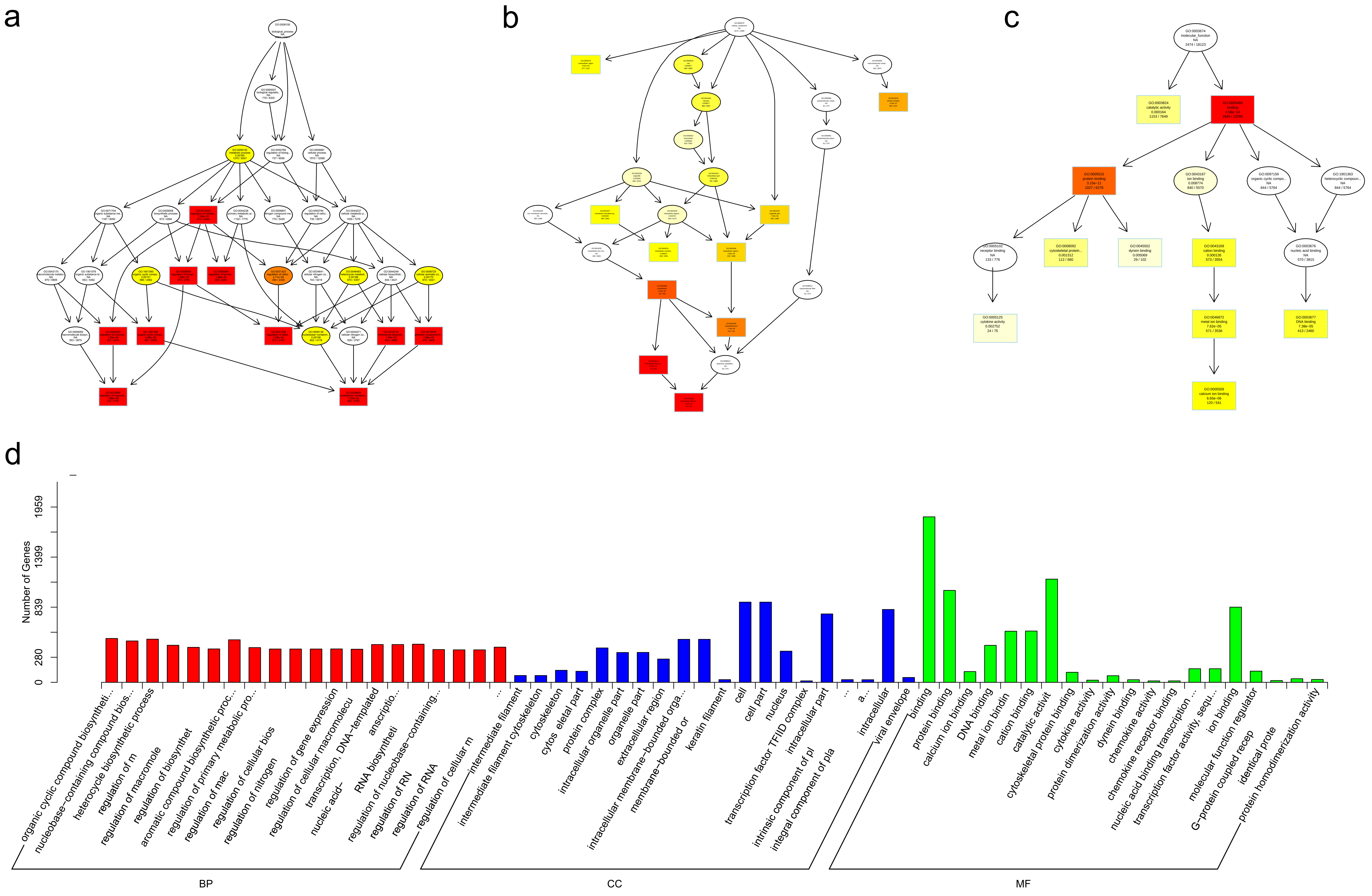

Figure S5. GO analysis of lncRNA-miRNA target mRNAs. (a–c) The significant molecular function, biological process and cellular component were enriched by lncRNA-miRNA-mRNAs in goat skins, and the DAG is the graphical display of GO enrichment results with candidate targeted genes. **(d)** The number of genes in GO terms were showed in histograph .

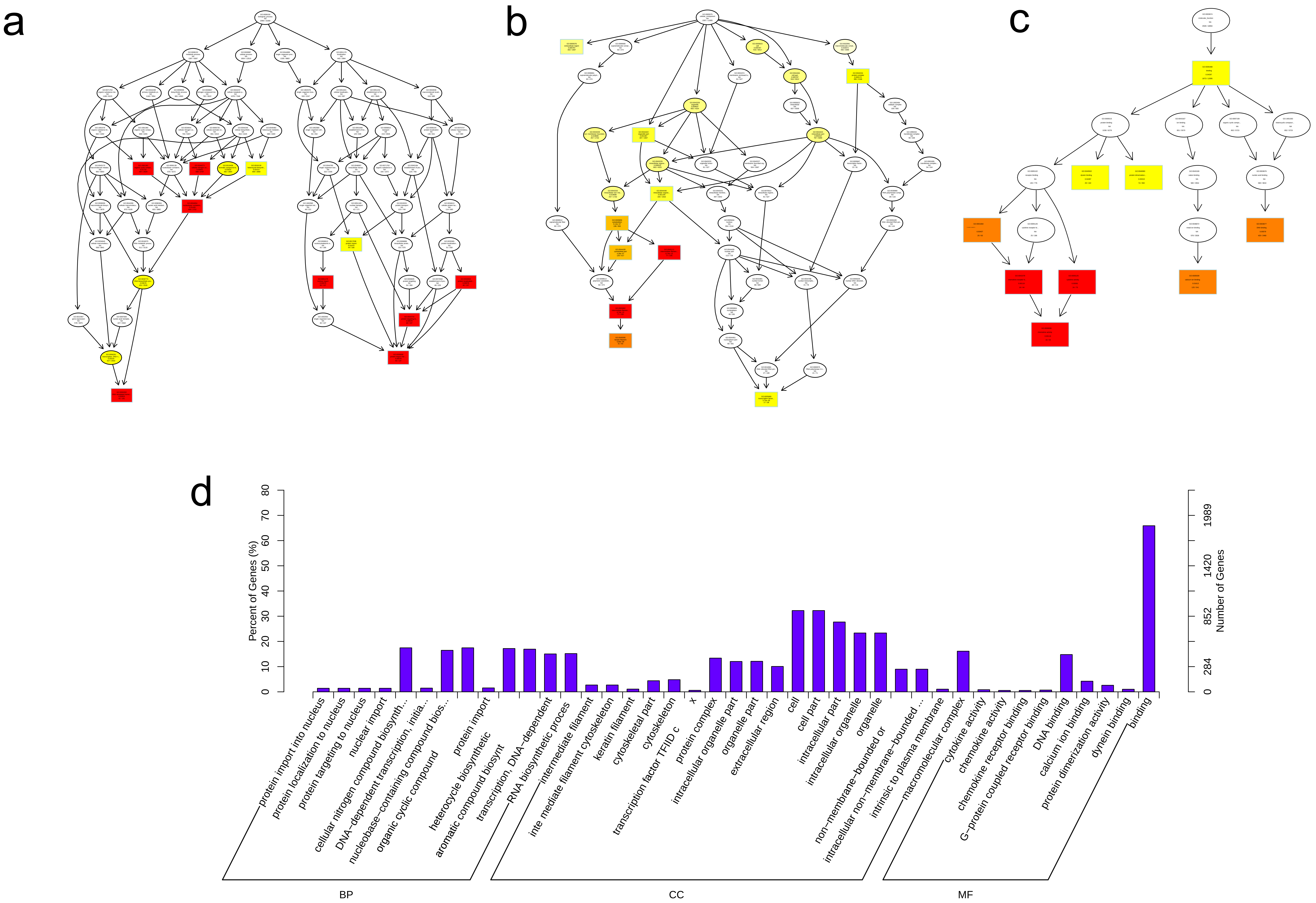

Figure S6. GO analysis of DE genes. (a–c) The significant molecular function, biological process and cellular component were enriched by DE mRNAs in goat skins, and the DAG is the graphical display of GO enrichment results with candidate targeted genes. **(d)** The number of genes in GO terms were showed in histograph .

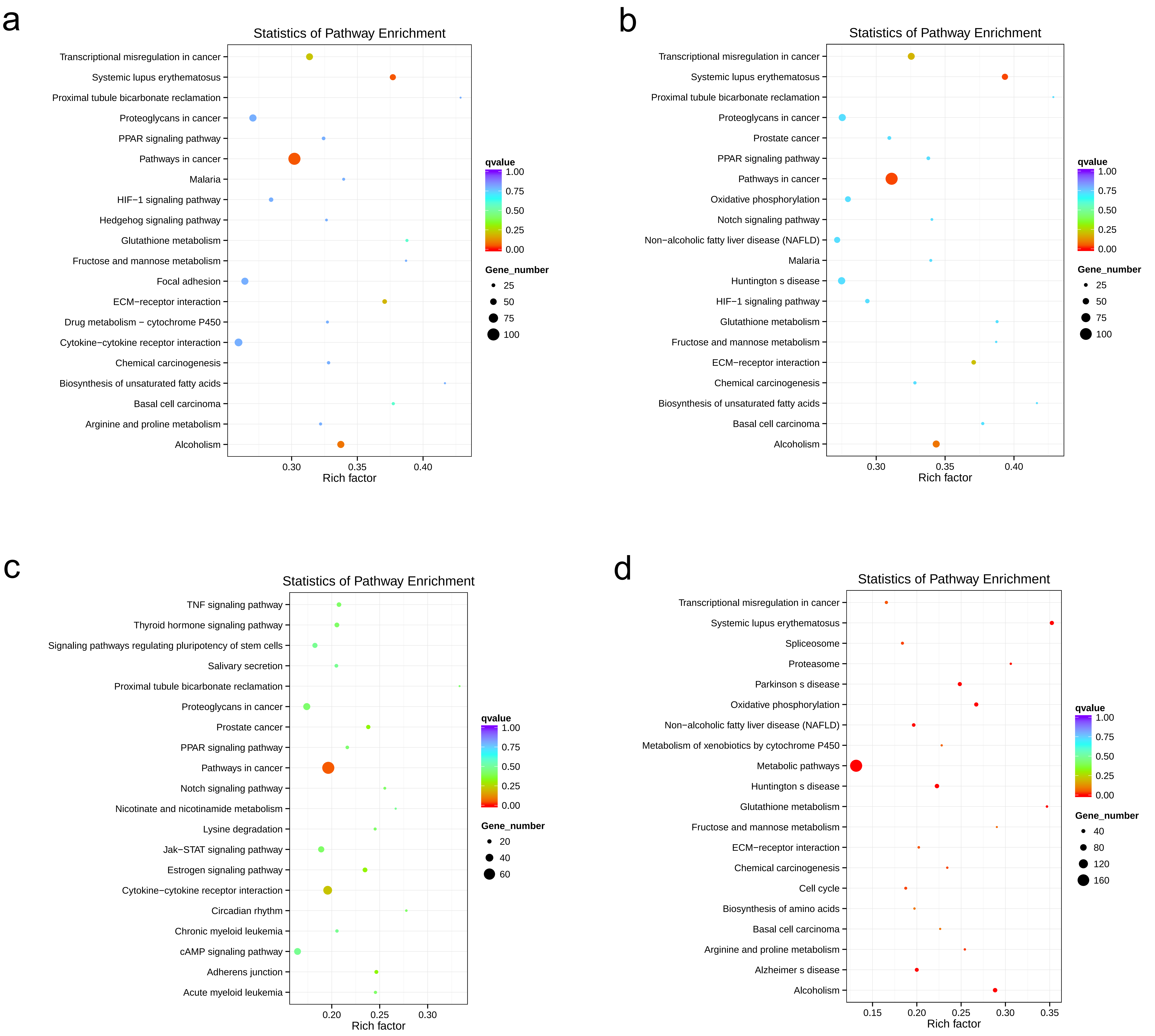

Figure S7. The top 20 KEGG pathways of hair cycle in skin. When the rich factor is greater, the Q-value is closer to zero, and the number of genes is greater, then the enrichment is more significant.
